# The Growth Promotion of Peppers (*Capsicum annuum* L.) by *Trichoderma guizhouense* NJAU4742-Based Biological Organic Fertilizer: Possible Role of Increasing Nutrient Availabilities

**DOI:** 10.3390/microorganisms8091296

**Published:** 2020-08-25

**Authors:** Qiumei Liu, Xiaohui Meng, Tuo Li, Waseem Raza, Dongyang Liu, Qirong Shen

**Affiliations:** Jiangsu Provincial Key Lab of Solid Organic Waste Utilization, Jiangsu Collaborative Innovation Center of Solid Organic Wastes, Educational Ministry Engineering Center of Resource-Saving Fertilizers, Nanjing Agricultural University, Nanjing 210095, Jiangsu, China; 2016203055@njau.edu.cn (Q.L.); 2015203032@njau.edu.cn (X.M.); 2018103144@njau.edu.cn (T.L.); 2018103120@njau.edu.cn (W.R.); shenqirong@njau.edu.cn (Q.S.)

**Keywords:** solid-state fermentation, sporulation, biological organic fertilizer, plant growth promotion, soil enzyme activity

## Abstract

*Trichoderma* spp. is a cosmopolitan group of soil fungi which plays a remarkable role in stimulating plant growth after interacting with plant roots and has good application prospects in intensive agriculture. In this study, rice straw and amino acids improved the population of *Trichoderma guizhouense* NJAU4742 under solid-state fermentation and helped us develop a new type of organic fertilizer. The effects of this biological organic fertilizer were evaluated in the growth of peppers (*Capsicum annuum* L.) for two seasons under sandy and mountain soils. In the first season, the yields in T6 (0.06% solid fermentation products in soil) and AT6 (added 0.06% solid fermentation products and 1% amino acid organic fertilizer in soil) treatments were increased by 41.8% and 52.3% in sandy soil and by 51.6% and 46.5% in mountain soil, respectively, compared with chemical fertilizer. During the second season, the same trend was obtained in both sandy and mountain soils. Soil peroxidase activity (125.2 μmol·g^−1^ dw), urease activity (58.7 μmol·g^−1^ dw) and invertase activity (13.11 mg·g^−1^ dw) reached their highest levels in biological organic fertilizer compared to the treatments with chemical fertilizer and solid fermentation products. Redundancy analysis showed that crop yield was positively correlated with enzyme activities, soil organic carbon, total nitrogen, and available phosphorus. Thus, we demonstrated that NJAU4742-enriched biological organic fertilizer could accelerate the transformation of nutrients and promote pepper growth.

## 1. Introduction

*Trichoderma* spp. are cosmopolitan soil fungi that interact a great deal with surrounding roots, soils and organic materials, and are beneficial for agricultural activities by acting as biofungicides, bioremediating soils contaminated with metals or chemical wastes and eliciting plant development and defense [1,2]. Extensive research has shown that *Trichoderma* spp. fungi colonize in plant roots, frequently promote plant growth, increase nutrient availability and crop productivity by competing with other microorganisms, release nutrients, modify the rhizosphere, produce antibiotics, act against mycoparasites and secrete cell wall-degrading enzymes [3,4]. Chemical fertilizers and pesticides are indispensable elements for the promotion of crop growth and production in agriculture [5]; nevertheless, over-application of these chemicals induces successive negative effects, including the destruction of soil structure, acidification and reduction in nutrient use efficiency [6]. Different organic fertilizers produced from various agricultural wastes have recently been recommended because of abundant nutrients and high content of organic matter. Organic fertilizers can provide essential nutrients, improve crop productivity, increase plant photosynthesis and have beneficial residual effects on subsequent crops [7]. However, the nutrient supply efficiency of organic fertilizer is lower than that of chemical fertilizer, which has caused a reduction in applications of organic fertilizer, particularly for biological organic fertilizer (BOF) [8]. Most BOFs are composed of *Bacillus* spp., *Pseudomonas* spp. and *Trichoderma* spp., which can not only promote plant growth and increase nutrient use efficiency but also inhibit soil-borne disease [9].

*Trichoderma* spp. play a vital role as plant growth-promoting fungi (PGPF), which can form biofilms and reestablish the microbial community in the rhizosphere with complex mechanisms, such as bio-fertilization, photo-stimulation and bio-control [10]. One of the prominent plant growth-promoting mechanisms of *Trichoderma* spp. is the secretion of secondary metabolites, including indole acetic acid (IAA), ethylene (ET), gibberellins (GAs), abscisic acid (ABA), phenolics, amino acids and sugars [11]. Many studies revealed that *Trichoderma* spp. can effectively dissolve insoluble or slightly soluble minerals (Fe_2_O_3_, MnO_2_, Zn and phosphate rock) in soil by acidifying the rhizosphere soil and increasing oxidase activity [12,13,14,15]. *Trichoderma* spp. produce Fe^3+^ chelating siderophores [4], which are subsequently taken up by the plant or absorbed as an Fe^3+^ phytosiderophore [16]. *Trichoderma* spp. can also improve carbon and nitrogen cycling in soil [17]. Wu et al. [18] reported that PGPF could mediate soil biochemical transformations of organic matter, mineralization of soil nutrients and nutrient fixation, which primarily depend on microbe-derived ecosystem functions. Nutrient absorption efficiency is driven by PGPF because they secrete various extracellular enzymes into the soil to decompose different complex organic polymers into soluble fractions, which are transported into the interior and then metabolized, resulting in the activation of extracellular mineral elements [19].

Fertilizer containing free amino acids has been widely used for promoting plant growth and improving plant quality, and it also can reduce the need for applications of various pesticides and protect the environment [20]. Besides, Lima et al. [21] found that the application of rapeseed cake, soybean meal or other meals with high contents of proteins as the fertilize agents could increase the amount and activity of microbes in soil. However, it was unrealistic to use these agricultural wastes as raw material for organic fertilizer production with the rise in the price. Thus, other protein-rich waste materials are getting more and more attention. Liu et al. [22] pointed out that amino acids hydrolyzed from animal carcasses were good additives for the production of biological organic fertilizer. With the continuous expansion of intensive cultivation, the amount of slaughterhouse waste which would lead to serious environmental pollution without reasonable and effective treatment increased sharply. In order to realize the resource recycling of slaughterhouses wastes, diluted sulfuric acid was used to hydrolyze the slaughterhouse waste to obtain an amino acid solution, which could also improve the effect of *Trichoderma* spp. on crop growth promotion.

Considering a longer growing season, the state of high nutrient contents in shoots but low transfer rates to peppers must be retained; that practice requires more irrigation and consumes a larger amount fertilizer than other crops. For a kilogram of yield, approximately 3.5–5.4 g of nitrogen (N), 0.8–1.3 g of phosphorus pentoxide (P_2_O_5_) and 5.5–7.2 g of potassium oxide (K_2_O) are required, and the absorption ratio is 1:0.2:1.3 [23]. Meanwhile, some mycorrhizal fungi and *Trichoderma* spp. could promote pepper growth, improve fruit quality, increase the relative contents of P and K in shoots and induce resistance to pepper diseases [24,25].

The objective of this study was to reveal the plant growth-promoting mechanisms of the NJAU4742-based-BOF (via improving the soil nutrient concentrations after application), which was prepared by optimizing the nutritional conditions for the sporulation of NJAU4742.

## 2. Materials and Methods

### 2.1. Preparation of T. guizhouense NJAU4742-Based Solid Microbial Spawn and Biological Organic Fertilizer 

*T. guizhouense* NJAU4742 (GenBank accession number: GQ337429) isolated from compost was stored in our laboratory, and the spores of this strain were preserved in 30% glycerin at −80 °C to prevent spawn degeneration. The *T. guizhouense* NJAU4742-based solid microbial spawn was prepared as follows: air-dried rice straw collected from Huai’an, Jiangsu Province was chopped into small fragments 50 mm in length, and ammonium sulfate and an amino acid solution obtained from hydrolyzing various animal hairs (provided by Jiangyin Lianye Biology Co., Ltd., Jiangyin City, China, with the fundamental characterization shown in Table S1) were applied separately to optimize the sporulation conditions for NJAU4742. Sterile water was used as the control. Twenty percent (v/w) amino acid solution and ammonium sulfate with an equal quantity of nitrogen were added into the rice straw; all ingredients were mixed well (Soak overnight); and then all substrates were adjusted to pH 3.5 and 75% water content. Five percent (v/w, 1 mL suspension per gram dry weight of substrate) fresh conidia suspension (1 × 10^7^ conidia·mL^−1^) of NJAU4742 was inoculated into the substrates, and the top of the vessel was sealed with cling film to prevent the water from evaporating too quickly. Five tiny openings were made above the parafilm to provide sufficient oxygen for microbial growth and all boxes were incubated at 28 °C. After 7 days, the quantification of spores was conducted as follows: approximately 10 g of the fermentation products was shaken at 180 rpm for 30 min in 100 mL of sterile water (1:10, w/v) at room temperature; the solid fractions and mycelium were removed by filtration with three layers of sterile gauze; and finally, the supernatants were used to quantify spore production. The NJAU4742-based BOF was obtained by adding the solid spawn with different concentrations of spores, and the specific production process was as follows: twenty percent amino acid solution (v/w) was added into mature cow manure compost, and different concentrations of solid spawn (1%, 3% and 6%, w/w) were blended fully and distributed evenly within the composts until the pH recovered to 5.0. After this process, the spore production was quantified in each treatment, according to the method as described above. BOFs were stored at room temperature for subsequent use. 

### 2.2. Soil Collection and Greenhouse Pot Experiment

Two types of soil (mountain soil and sandy soil) were used in the pot experiment. The characteristics of the original soil are shown in Table S2. The mountain soil (viscous) was sampled from the permanent meadow (more than 60 years) at Yixing, Jiangsu Province, which is deficient in nitrogen, phosphorus, alkalis and alkaline earth metals but abundant in iron and aluminum oxide. The sandy soil (arenosol) was sampled from Haian, Jiangsu Province, which was cultivated with maize cultivation with soybean rotation for more than 10 years. The sandy soil was characterized by abundant humus, loose texture, high fertility and porosity ratio and high permeability. The pot experiment was conducted in a greenhouse in Yixing, Jiangsu Province. The average temperature of the area is 28 °C, and the rainfall (annual average rainfall of 1177 mm) primarily occurs in summer and autumn. 

The prepared solid spawn (with more than 10^10^ g^−1^ dw conidia of NJAU4742) and BOF were mixed with the air-dried soils, and the treatments were as follows: T0: 10 g·kg^−1^ (w/w, dw) chopped rice straw with the size of 1–2 mm; T1: 10 g·kg^−1^ (w/w, dw) solid spawn; T3: 30 g·kg^−1^ (w/w, dw) solid spawn; T6: 60 g·kg^−1^ (w/w, dw) solid spawn; AA: 200 mL·kg^−1^ (v/w, dw) amino acid solution; AT1: 200 mL·kg^−1^ (v/w, dw) amino acid solution and 100 g·kg^−1^ (w/w, dw) BOF; AT3: 200 mL·kg^−1^ (v/w, dw) amino acid solution and 300 g·kg^−1^ (w/w, dw) BOF; AT6: 200 mL·kg^−1^ (v/w, dw) amino acid solution and 600 g·kg^−1^ (w/w, dw) BOF; BT: the *Bacillus amyloliquefaciens* SQR9-based BOF we produced (stored in our laboratory); CK: chemical fertilizer. All treatments contained equal concentrations of total nutrients, whose values are shown in Table S3. Pepper seeds (*Capsicum annuum* L.) used in this study were Sujiao No.1 obtained from the Jiangsu Academy of Agricultural Sciences. The seeds were disinfected with 70% (v/v) ethanol for approximately 1 min and then dipped into 20% sodium hypochlorite (NaClO) for 20 min, after which the seeds were rinsed with sterilized water at least four times. The sterilized pepper seeds were placed in darkness at 30 °C for 36 h on a sterile double layer of filter paper to ensure humidity for seed germination, and then the viable seeds were cultivated in nursery substrate for approximately 15 days. Well grown pepper seedlings were transplanted into different pots (200 × 180 mm, two seedlings in each pot) containing 3.2 kg of soil, with six replicates for each treatment. Different treatments were randomly distributed to avoid the influences of environmental factors; they were all given 15 h of light (21–28 °C) and 9 h of darkness (10–15 °C) per day, and the watering was arranged according to crop requirements.

Sampling was conducted at different growth stages: during the transition period of the vegetative growth stage, when differences between treatments emerged; and during the maturation stage, when there were significant differences in the fruits between treatments). Three pots of each treatment were sampled randomly when a significant difference in the pepper shoots among different treatments was detected; the remaining three pots of each treatment were sampled during the reproductive growth stage (termination of fruit production stage). The rhizosphere soil samples were collected: Roots were placed horizontally on the experimental rig and shaken vigorously to separate bulk soil, and then approximately 5 g of soil was collected via spatula within 1 mm of the taproot and lateral roots; the regions surrounding the root tips were avoided for use in subsequent experiments. Bulk soils were also collected by the quartering method and then used as the samples to determine various physical and chemical properties. The roots were put into different conical flasks containing 45 mL of sterile water and then kept on a shaker at 180 rpm at 28 °C for 30 min. The suspensions were centrifuged at 6000 rpm at 4 °C for 10 min, and the precipitates considered the rhizosphere soil were preserved at −80 °C immediately after discarding the supernatants for the subsequent experiment. At the same time, soil samples were collected at the depth of 5–10 cm. 

### 2.3. Determinations of Pepper Biomass and the Soil’s Physicochemical Properties

Various parameters of each pepper, such as shoot length, shoot diameter, shoot dry weight, root dry weight and leaf area were measured during the vegetative period. Parameters of fruit quality, including concentrations of vitamin C (VC), soluble sugars, proteins and nitrate were determined. Soil physical and chemical properties were analyzed. Soil available phosphorus (AP) was extracted with 0.5 M NaHCO_3_ and then determined using the ammonium molybdate ascorbic method. Soil organic C (SOC) and total N (TN) were determined using a TOC analyzer (Elementar, Langenselbold, Germany). Soil pH was assayed in a soil extraction solution (1:2.5, w/v) with a compound electrode (PE-10; Sartorious, Göttingen, Germany). Available potassium (AK) was extracted with 1 M ammonium acetate solution and then determined using a flame spectrophotometer (FP640: INASA, Shanghai, China). NO_3_^−^-N and NH_4_^+^-N were extracted with 0.01 M CaCl_2_ and the contents were determined with a continuous flow analytic system (Santt System; Skalar, Holland).

### 2.4. Quantitative Estimation of Different Culturable Microorganisms

Five grams of rhizosphere soil was transferred into a conical flask containing 45 mL of sterile water and then placed on a shaker at 30 °C and 180 rpm for 30 min. Quantitative estimation of various culturable aerobic microorganisms, including filamentous eumycetes, bacteria and actinomycetes, was conducted by inoculating the appropriate media with 0.1 mL volumes of different ten-fold serial dilutions. Media and incubation times for different microorganisms were as follows: nutrient agar for 2 days for aerobic bacteria; sodium caseinate agar for 4 days for actinomycetes; and rose bengal agar for 4 days for aerobic filamentous eumycetes. The quantification of *Trichoderma* spp. in the rhizosphere soil of different treatments was conducted. A 100 µL aliquot of soil suspension that was serially diluted to 10^−4^, 10^−5^ and 10^−6^ was uniformly spread on a plate containing *Trichoderma* spp. selective medium [26] and placed in a stable incubator at 28 °C for 2 days. The colonies on the plates were counted, and the results were expressed as colony-forming units (CFU) per dry weight of soil.

### 2.5. Soil Total DNA Extraction and Quantitative Determinations of Different Microorganisms via Fluorescence

DNA was extracted using a MOBIO Power Soil™ DNA Isolation Kit (MO BIO Laboratories, Carlsbad, CA, USA) according to the manufacturer’s instructions, and total DNA solutions were further cleaned with a Wizard® PCR Preps DNA Purification System kit (Promega, Madison, WI, USA) to remove other contaminants. DNA concentration was measured with a Qubit® fluorometer with Quant-iT Qubit dsDNA BR assay kit (Invitrogen™, Thermo Fischer Scientific, Waltham, MA, USA). From the quantitative data obtained from the fluorescent-labeled DNA fragments, the number of copies was obtained. PCR was carried out using a 7500 Fast Real-Time PCR system (Applied Biosystems, Waltham, MA, USA), and all primers shared the same amplification: an initial step of 6 min at 95 °C, followed by 40 cycles of 95 °C for 30 s, 60 °C for 1 min, a plate reading step and the product melting curve of 55 °C to 95 °C. The conserved regions of total fungi, total bacteria and NJAU4742 were amplified by the PCR with primers, and the nucleotide sequences of the primers 515F/907R, ITS5/ITS4 and *Trichoderma* strain-specific primers are listed in Table S4 [27,28,29]. The PCR mixture contained: 1 µL DNA templates, 7.5 µL SYBR Premix Ex Taq (28×), 0.4 µL ROX reference dye II (50×) (Takara, Dalian, China) and 0.45 µL each microbe-specific primer for a total volume of 20 µL. The standard curve was obtained through culturing a single clone of the target gene fragment and extracting the plasmid; selecting 6 appropriate concentration gradients for quantitative PCR reaction; and drawing the standard curve. The gene copy number of the target microbial group was calculated from the standard curve based on the Ct value of each sample, and the results were expressed as the logarithmic values of the numbers of copies per gram of soil (log^10^ copies g^−1^ soil). The results are presented as means ± standard deviations of three replicates. 

### 2.6. Soil Enzyme Activity Determinations

Various hydrolytic enzymes relating to the transformations of carbon, nitrogen and phosphorus were determined. Invertase activity was determined by the 3,5-dinitrosalicylic acid method, and the developed color was measured at 508 nm by a Multi-Detection Microplate Reader (SpectraMax M5; Molecular Devices, San Jose, CA, USA). Urease activity was determined by the indophenol colorimetric method, and one unit of urease activity was expressed as the concentration of enzyme required to release 1 mg of ammonia per day for 1 g soil under the above assay. Determination of acid phosphatase and peroxidase activity was done by the fluorescence intensity of 4-methylumbelliferone (MUB) released from 200 Um 4-MUB phosphate and 5 mM L-3, 4-dihydroxyphenylalanine (L-DOPA; Sigma-Aldrich Co. Ltd., Dorset, UK) after hydrolysis by the soil crude enzymes. Two grams of fresh soil was placed in an Erlenmeyer flask containing 100 mL of 0.5 mol·L^−1^ (pH 5.5) acetic acid buffer and then shaken at 30 °C for 60 min; 100 μL aliquot of soil suspension and a 100 μL aliquot of substrate A/B were added to a 96-well plate, after which the plate was kept in a dark incubator at 30 °C. The incubation times for the phosphatase and peroxidase activities were 14 and 24 min, respectively, and then 100 μL of 2 mol·L^−1^ NaOH was added to stop the reactions when the fluorescence intensity of the sample reached half that of the MUB quench standard. The fluorescence intensity assay was performed using a multifunctional microplate reader (SpectraMax M5; Molecular Devices, San Jose, CA, USA), and the excitation and emission wavelengths were set as 365 and 470 nm, respectively. One unit of acid phosphatase activity and peroxidase activity was expressed as μmol substrate transformed by one gram of oven-dry soil equivalent per day.

### 2.7. Statistical Analysis

Linear correlation analysis was used to evaluate the correlations between various parameters of soil (hydrolytic enzyme activities, nutrient contents and yields) using R software (version 3.3.3). The redundancy analysis (RDA) was conducted using Canoco (version 5.0), and the results of RDA are presented graphically in the bi-plots. Relationships (inverse or directly proportional) of different vectors were interpreted based on the positions of the parameters in terms of arrow ends. Data were expressed as the means of three replicates and were analyzed by using one-way ANOVA using SPSS 16.0 software, and the LSD method was used to study the significance of treatment (*p* < 0.05). Correlation analysis was performed by using R software (version 3.3.3), and RDA was analyzed by Canoco (version 5.0). 

## 3. Results

### 3.1. Preparation of Solid Spawn and T. guizhouense NJAU4742-Based Biological Organic Fertilizer

**Figure 1:**
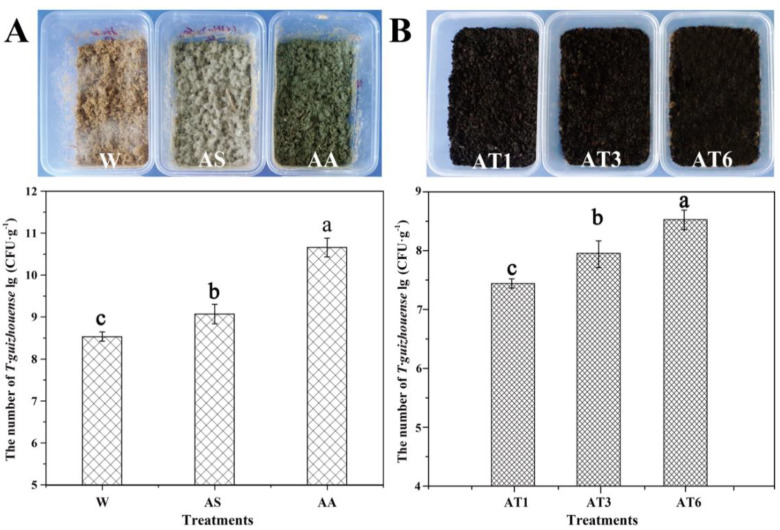
Preparation of *Trichoderma guizhouense* NJAU4742 solid spawn (**A**) and NJAU4742-based biological organic fertilizer (**B**). W means sterile water, AS indicates ammonium sulfate, AA is the treatment fortified with amino acid solution; AT1: 20% (v/w, dw) amino acid solution and 1% (w/w, dw) biological organic fertilizer (BOF) (w/w, dw); AT3: 20% (v/w, dw) amino acid solution and 3% (w/w, dw) BOF; AT6: 20% (v/w, dw) amino acid solution and 6% (w/w, dw) BOF. Data are repressed as means ± SDs of 3 replicates. Values with different letters in the same column are significantly different at *p* ≤ 0.05 according to Duncan’s ANOVA test.

The NJAU4742 solid spawn was produced using rice straw as the substrate under solid-state fermentation (SSF), and ddH_2_O (W), ammonium sulfate (AS) and amino acid (AA) solutions were added to optimize the sporulation of NJAU4742. Amino acids significantly promoted the sporulation of NAJU4742, which was better than that in the AS treatment, whereas no obvious sporulation occurred in the W treatment (Figure 1A). The numbers of spores quantified by gradient dilution count were 3.46 × 10^6^, 1.19 × 10^9^ and 4.62 × 10^10^ in W, AS and AA treatments, respectively. The results indicated that the rice straw was a suitable carbon source for spore production by NJAU4742. The additions of ammonium sulfate and the amino acid solution increased the production of spores by 3 and 4 fold, respectively, compared with that of the CK. Different quantities of solid spawn obtained from SSF were added into matured compost to form the NJAU4742-based BOF, and after thorough mixing, the number of NJAU4742 in each sample was quantified (Figure 1B). The numbers of NJAU4742 in AT1, AT2 and AT3 were 2.73 × 10^7^, 9.04 × 10^7^ and 2.03 × 10^8^, respectively. 

### 3.2. Effects of Solid Spawn and T. guizhouense NJAU4742-Based BOF on Pepper Growth

**Figure 2:**
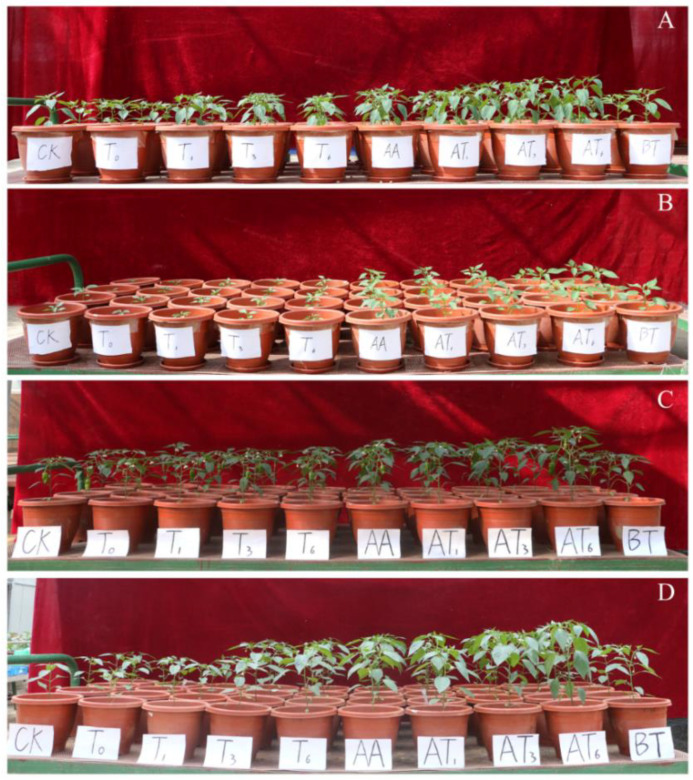
The pepper growth promotion in different treatments in sandy soil and mountain soil during two growth seasons. (**A**) The pot experiment results in sandy soil during the first season (FS); (**B**) the pot experiment results in mountain soil during the FS; C) the pot experiment results in sandy soil during the second season (SS); D) the pot experiment results in mountain soil during the SS; the treatments of the greenhouse pot experiment were as follows: T0: adding 0.01% (w/w, dw) chopped rice straw with the size of 1–2 mm; T1: 0.01% (w/w, dw) solid fermentation products; T3: 0.03% (w/w, dw) solid fermentation products; T6: 0.06% (w/w, dw) solid fermentation products; AA: 20% (v/w, dw) amino acid solution; AT1: 20% (v/w, dw) amino acid solution and 1% (w/w, dw) BOF (w/w, dw); AT3: 20% (v/w, dw) amino acid solution and 3% (w/w, dw) BOF; AT6: 20% (v/w, dw) amino acid solution and 6% (w/w, dw) BOF; BT: *Bacillus amyloliquefaciens* SQR-9-based BOF (stored in our lab); CK: chemical fertilizer.

The effects of solid spawn and *T. guizhouense* NJAU4742-based BOF on pepper growth promotion were evaluated in a pot experiment (Figure 2). The results indicated that different quantities of NJAU4742 in solid spawn and biological fertilizer could promote the growth of pepper in both types of soil, and the promotion of pepper growth in sandy soil was better than that in the mountain soil during the two seasons. However, the growth promoting effects of BOF treatments on pepper were better than those of solid spawn treatments in both soils during the two seasons.

As shown in Table 1, the shoot height, stem diameter, chlorophyll content, leaf number, plant weight, root weight and vitamin C content of pepper were increased with the increase in the numbers of NJAU4742 contained in the agents, particularly the root dry weight, which was significantly higher than that of the CK in both soils. In sandy soil, the root dry weights of T6 and AT6 were increased by 16.2% and 70.0%, and in the mountain soil, by 26.7% and 113.3%, respectively, compared with the CK. The shoot heights of T6 and AT6 were increased by 1.57% and 47.79%, respectively, compared with the CK during the first season (FS). The highest VC content was obtained in AT6, whereas the lowest VC content was found with the CK in both soils during the two seasons, indicating that the BOF could also improve the quality of pepper. The yields of different treatments during the two seasons in the two soils are shown in Table 2. In the mountain soil, the yields of T6 and AT6 treatments were increased by 51.6% and 146.5%, respectively, in the FS, whereas yields were increased by 51.9% and 78.2%, respectively, during the second season (SS), compared with CK. Pepper growth was promoted by the applications of *Trichoderma*-based solid spawn and BOF to the sandy and mountain soils, although the growth-promoting effect of BOF treatment was even better than that of solid spawn.

### 3.3. Determination of Soil Enzyme Activities

**Figure 3:**
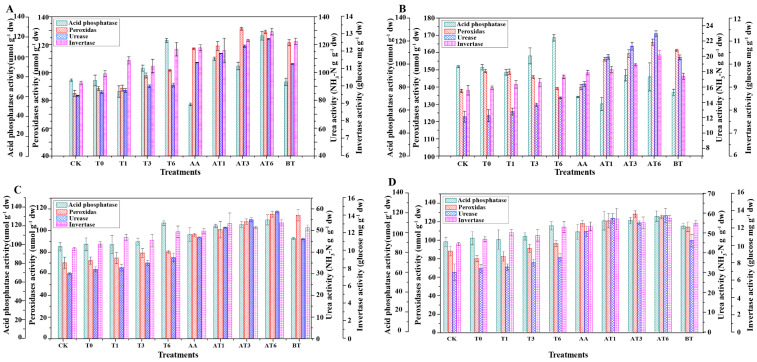
The soil’s extracellular enzyme activities, including invertase, urease, peroxidase and acid phosphatase in sandy soil and mountain soil during two growth seasons. (**A**) the enzyme activities of different treatments in sandy soil during the FS; (**B**) the enzyme activities of different treatments in sandy soil during the SS; (**C**) the enzyme activities of different treatments in mountain soil during the FS; (**D**) the enzyme activities of different treatments in mountain soil during the SS.

The activities of soil acid phosphatase, peroxidase, urease and invertase are shown in Figure 3. Figure 3A shows the enzyme activities of different treatments in sandy soil during the FS. The highest acid phosphatase activity was obtained in T6, whereas the maximum peroxidase activity, urease activity and invertase activity were detected in AT6. No difference was detected in acid phosphatase activities between T6 and AT6. Maximum peroxidase activity, urease activity and invertase activity in AT6 are shown in Figure 3B. Figure 3C shows the enzyme activities of different treatments in sandy soil during the SS. All enzyme activities in BOF treatments were generally higher than those of solid spawn and the CK. The highest peroxidase activity was obtained in the AT3 treatment; however, no significant differences were detected among AT1, AT3 and AT6. Figure 3D shows the enzyme activities of different treatments in the mountain soil during the FS, and compared with sandy soil, similar results were obtained in the mountain soil. The maximum acid phosphatase activity was obtained in T6. The activities of peroxidase and urease in treatments of BOF were higher than those in other treatments.

### 3.4. Contents of Total Nitrogen, Soil Organic Carbon and Available Phosphorus in Soil

The soil’s physiochemical properties, including SOC, TN and AP are shown in Table 3 (sandy soil) and Table 4 (mountain soil). The contents of SOC, TN, AP and AK in T6 of sandy soil during the FS were significantly increased by 10.3%, 6.28%, 10.09% and 22.03%, respectively, compared with those in the CK, whereas those increases were 22.28%, 16.74%, 122.62% and 10.42%, respectively, in AT6 compared with those in the CK of sandy soil during the SS. The values of most physiochemical properties in the sandy soil during the SS were higher than those of the FS, except the contents of NO_3_^−^ and AP. The results also showed that the NO_3_^−^ contents of the treatments with amino acids were decreased more rapidly than those of other treatments, particularly for AT6, in which the NO_3_^−^ content was decreased by 85.9% compared with that in the FS. The results showed that the pH in sandy soil remained relatively constant, and the highest pH value was 7.69 in the AA treatment during the second season.

The physiochemical properties in the mountain soil during the two seasons in different treatments are shown in Table 4. The highest contents of SOC, TN, NH_3_^+^, AP and AK were obtained in AT6, except NO_3_^−^, with the increases of 31.30%, 17.95%, 130.59%, 122.59% and 26.85%, respectively, compared with those of the CK. The content of NO_3_^−^ was gradually increased in the mountain soil during the two seasons, which was a different response than that in sandy soil, and the maximum NO_3_^−^ content was obtained in the CK. When compared with the contents of different nutrients in AA during the FS, SOC, TN, AP, and AK in AT6 were increased by 7.4%, 24.2%, 12.0% and 15.8%, respectively.

### 3.5. Correlation Analyses of Soil Physiochemical Properties, Soil Enzyme Activity and Pepper Yield

**Figure 4:**
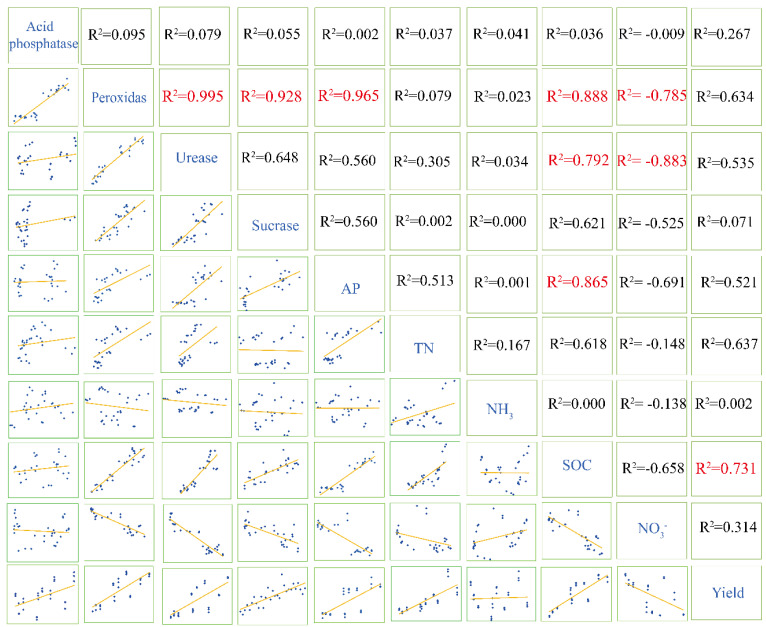
The linear correlation analysis among various parameters containing enzyme activities, physiochemical characteristics, and yields; * means the correlation is significant at the 0.05 level, and ** indicates that correlation is significant at the 0.01 level.

Linear correlation analysis was performed to evaluate the correlations among hydrolytic enzyme activities, nutrient contents, and pepper yield in different treatments (Figure 4). The relations between different parameters were evaluated by linear correlation analysis in the form of the slope and distribution. The results showed that peroxidase activity had a clear and consistent positive correlation with urease activity (r = 0.995, *p* < 0.01), invertase activity (r = 0.928, *p* < 0.01), AP (r = 0.965, *p* < 0.01) and SOC (r = 0.888, *p* < 0.05). However, the correlation was negative between the peroxidase activity and the content of NO_3_^−^ (r = −0.785, *p* < 0.05). The correlation was positive between urease activity and SOC (r = −0.785, *p* < 0.05), whereas a negative correlation was found between urease activity and content of NO_3_^−^.

**Figure 5:**
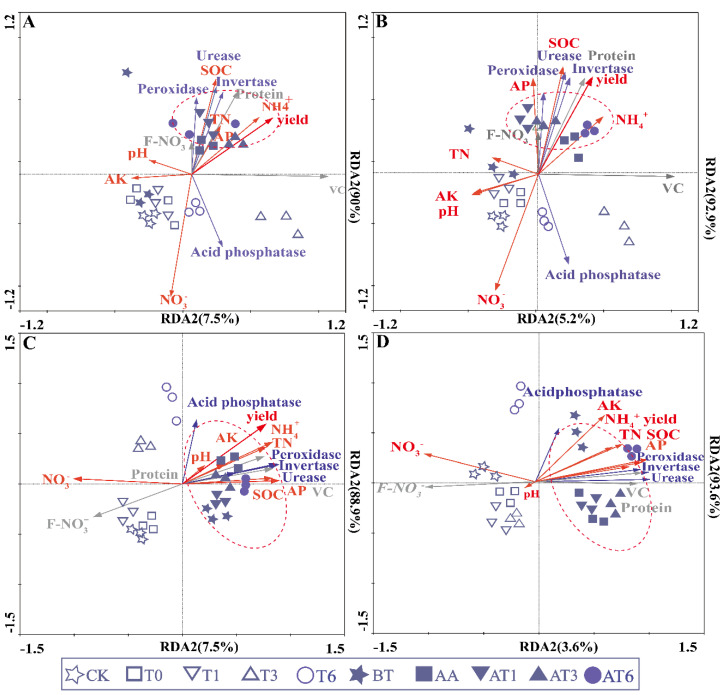
The correlations of different parameters, including soil physicochemical properties, soil enzyme activities and yields. SOC means soil organic carbon; TN means total nitrogen; AP means available phosphorus; AK means available potassium; F-NO_3_^−^ means content of NO_3_^−^ in fruit.

The RDA analysis of the pot experiments in the sandy soil of the FS and the SS is shown in Figure 5. Most environmental factors along axis 1 that increased (93.6% variation, 0.036 fitting variations) were positively correlated with SOC, TN, AP, pH and VC contents and negatively correlated with NH_3_^+^, NO_3_^−^ and F-NO_3_^−^ contents, and this axis was higher than that of any other variables, as indicated by the length and direction of the vector. The results showed lesser correlations among soil enzyme activity, TN, NO_3_^−^, AP, SOC, AK, pH and yields, which indicated a positive correlation between soil enzyme activity and other parameters. Additionally, the corner dimension showed that both soil enzyme activity and TN were positively correlated with yield. The arrow of AK was the shortest one of all parameters, indicating the minimum correlations were between AK and various soil enzyme activities. Fruit quality also presented a notable positive correlation with various soil enzyme activities. On the first axis, AT6, AT3, AT1 and AA were positively correlated with most of the enzyme activities, physicochemical factors and yields, and the correlation order was as follows: AT6 > AT3 > AT1 > AA. However, the results showed low correlations of T6, T3, T1, T0 and the CK with enzyme activities, physical and chemical factors and yields compared with those of BOF treatments. These results illustrated that the transformations of carbon and nitrogen in soil were closely correlated with enzyme activities by the application of NJAU4742-based BOF.

### 3.6. Quantification of Rhizosphere Microorganisms

**Figure 6:**
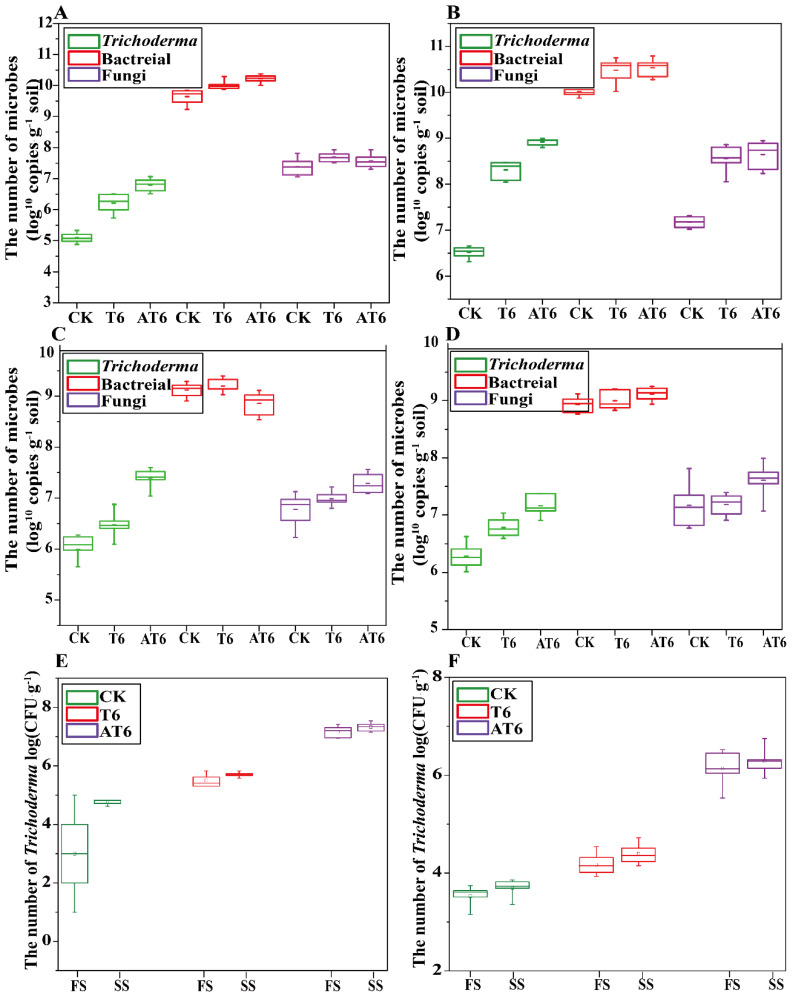
The quantification of total bacteria, fungi, and *Trichoderma* spp in the plant root rhizospheres of different treatments. (**A**,**B**) are the quantification results of total bacteria, fungi and *Trichoderma* spp. in sandy soil during the FS and SS, respectively; (**C**,**D**) are the quantification results of total bacteria, fungi and *Trichoderma* spp. in sandy soil during the FS and SS, respectively; (**E**) the numbers of culturable *Trichoderma* spp. in CK, T6 and AT6 in the sandy soil during the FS and SS; (**F**) the numbers of culturable *Trichoderma* spp. in CK, T6 and AT6 in the mountain soil during FS and SS.

Microbial species and numbers in the plant rhizosphere among different treatments were compared with the role of NJAU4742-based BOF. The quantitative results of the rhizosphere total bacteria, fungi and *Trichoderma* spp. in the pot experiment in sandy soil during the FS and the SS are shown in Figure 6A and Figure 6B, respectively. The quantitative results indicated that the numbers of the detected microbes in CK, T6 and AT6 shared the same tendency (bacteria > fungi > *Trichoderma*) in sandy soil during the two seasons, and the numbers of bacteria, fungi and *Trichoderma* spp. gradually increased with the increasing number of NJAU4742 added into the soil. Treatment AT6 had the highest logarithm order number of fungi (7.44 in the FS and 8.65 in the SS) and *Trichoderma* spp. (6.72 in the FS and 8.72 in the SS); however, no significant differences were detected among CK, T6 and AT6 during the FS and SS. During the FS, the numbers of bacteria, fungi and *Trichoderma* spp. in AT6 were increased by 20%, 23% and 54%, respectively, compared with those in the CK, whereas they were increased by 2%, 5%, and 16% in T6 during the FS, respectively.

The numbers of bacteria, fungi and *Trichoderma* spp. in the mountain soil are shown in Figure 6C (FS) and Figure 6D (SS). The results indicated that the numbers of bacteria, fungi and *Trichoderma* spp. in each treatment of mountain soil were lower than those obtained in sandy soil during both seasons. The maximum logarithm order number (10.46, 8.92 and 8.61 during the FS and 7.42, 10.6 and 7.82 during the SS, respectively) of each microbe group was also obtained in AT6 during both seasons. The numbers of *Trichoderma* spp. in T6 and AT6 were increased by 2.4% and 20.1%, respectively, compared with that of the CK during the FS, whereas they increased by 5.87% and 13.0%, respectively, compared with that of the CK during the SS. No significant difference was detected for the bacterial number among the CK, T6 and AT6 in both seasons. The numbers of fungi in T6 and AT6 were increased by 5.61% and 8.23%, respectively, compared with that of the CK during the FS, and increased by 5.25% and 7.81%, respectively, compared with that of the CK during the SS. The number of *Trichoderma* spp. in the rhizosphere soil was also detected by the gradient dilution method to investigate the effective number of viable cells (Figure 6E (FS) and Figure 6F (SS)), and the results indicated that the highest number of *Trichoderma* spp. was obtained in AT6 in both seasons (6.96 in the FS and 7.42 in the SS), and the increases were 57.8% and 54.6%, respectively, compared with those of the CK during the two seasons.

**Figure 7:**
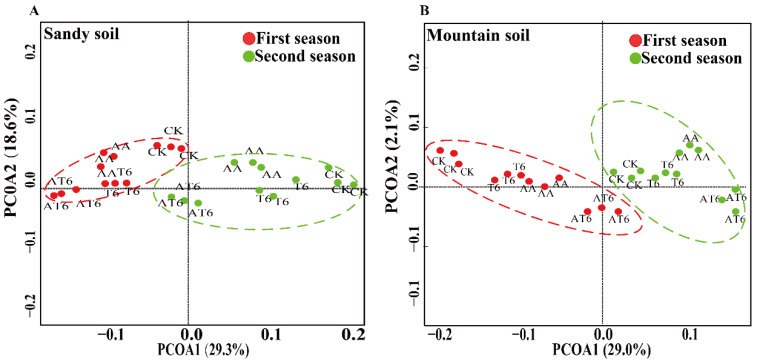
The PCoA analysis of different treatments in sandy and mountain soil during two growth seasons. (**A**) the results obtained in sandy soil, (**B**) the results obtained in mountain soil.

The microbial community structure among different treatments was analyzed by multivariate statistical PCoA (Figure 7), and the results showed that the treatment AT6 was primarily located in PC1 and was significant different to AA, T6 and the CK. Strikingly, significant differences were observed in the quantities of *Trichoderma* spp., bacteria and fungi between the treatments with chemical and organic fertilizers. Furthermore, the BOF treatments significantly increased the numbers of *Trichoderma* spp. and total fungi compared with those in other treatments with equal nutrients during both seasons.

## 4. Discussion

### 4.1. Pepper Growth Promotion by NJAU4742-Based Biological Organic Fertilizer

NJAU4742 could contribute substantially to green agricultural production by activating soil nutrients and promoting plant growth. Simultaneously, the exogenous addition of amino acids’ hydrolysates into the substrates provided an efficient way to break through the bottleneck of obtaining a large number of *Trichoderma* conidia, and the *Trichoderma*-based BOF could promote plant growth by increasing the soil nutrients’ availabilities, which was coincident to the hypotheses put forward in this study. To our knowledge, this is the first study to find that the amino acids’ hydrolysates from the slaughterhouses’ wastes can exert strong effects on the efficient spore production of *Trichoderma* spp. Large amounts of the fermented products of NJAU4742 promoted crop growth, achieved high crop yields and improved the transformation and effectiveness of nutrients in the soil. For instance, SSF has become an essential technology for the production of microbial products such as fertilizers, fuels, foodstuffs, feed-stuffs and medicines [30]. In this study, different agricultural wastes, including rice straw; amino acid solution from discarded poultry and livestock; and matured livestock manure were used as substrates to achieve a value-added approach for BOF production. The SSF results showed that NJAU4742 grew well and formed many conidia in the AA treatment (1.2 × 10^10^ CFU·g^−1^ dw), which greatly exceeded the numbers obtained in the CK and AS. This result might have been due to the application of the amino acids which were used by microbes as a food source. The slaughterhouse wastes’ hydrolysates contained abundant free amino acids which provided rich nutrients for NJAU4742, while the adversity from the low pH stress also resulted in an obvious promotion of sporulation [31]. Jian et al. [32] noted that the evolutions of fungal colonies, spore formation and metabolic activity were significantly affected by nutrition, oxygen, pH and temperature during SSF. Generally, the heat tolerance of *Trichoderma* spp. is poor; thus, a product of *Trichoderma* spp.-based BOF through a traditional method (second fermentation) such as that used for *Bacillus* spp.-based BOF is difficult to obtain.

Compared with indigenous microorganisms, exogenous microorganisms should overcome the difficulty of absorbing nutrients in the soil, thereby achieving a balance in the nutrients for crop growth, after which they could accelerate the transformation of nutrients [33]. The results of the pot experiment in sandy and mountain soils during two seasons showed that the beneficial microorganism NJAU4742 significantly promoted pepper growth, likely by increasing the photosynthetic intensity of the peppers, resulting in the accumulation of dry matter and increases in yields. The effects of plant growth promotion by *Trichoderma*-based BOF were better than those of the chemical fertilizer with equal nutrients, which is consistent with previous results [34]. Increases in plant height, stem diameter, stem weight, dry matter weight, yield and fruit quality were found in AT6 treatment in sandy soil. Similarly, the effects of BOF on plant growth promotion in mountain soil (with poor nutrient conditions) were better than those of the CK, which might be primarily attributed to the ability to promote root length, increase the number of root hairs to explore larger spaces of soil to absorb nutrients, transport more available nutrients, release organic acids and biosynthesize plant stimulatory compounds, such as growth hormones (indole acetic acid, cytokine, gibberellins and zeatin) [35]. The strain NJAU4742 significantly increased the root growth and the crop productivity when well colonized in the plant rhizosphere. These results could be due to contributions from increases in nutrient absorption, the effectiveness of various nutrients, biomass accumulation, transpiration rates of water, iron carrier production and mineral uptake that results from the interaction between the beneficial microorganism and the plant [36]. Hence, agricultural sustainability could be enhanced by *Trichoderma*-based biological organic fertilizer and management practices which could support the soil by replacing chemical fertilizer partially. However, it must be pointed out that the plant growth promotion effect of NJAU4742-based BOF was affected by various other parameters, including soil type, climates, land use, crop species and years. The results obtained by Ogle et al. [37] demonstrated that agricultural management impacts on SOC storage would vary depending on climatic conditions that influence the plant and soil processes driving soil organic matter dynamics. Moreover, Cai et al. [38] reported that the mineral-associated organic carbon/total soil organic carbon ratio was mainly driven by soil texture, soil types and related climate and land uses, and thus the effect of the *Trichoderma*-based BOF on the plant growth promotion was evaluated with two different soils in two seasons by pot experiments.

### 4.2. Soil Enzyme Activity and Nutrient Availability 

Various soil enzymes are involved in soil biochemical processes, including the synthesis and decomposition of humus; the hydrolysis and conversion of organic compounds of animal, plant and microbial residues; and oxidation-reduction reactions of organic and inorganic compounds in soils [39]. These processes are closely related to the release and storage of various nutrients, and the development and formation of humus structure/the physical conditions of soil [40]. The results indicated that the PGPR-based BOF increased soil enzyme activities, which would help NJAU4742 to resist many chemical reactions and activate soil nutrients and release organic matter into the soil, subsequently resulting an increase in the nutrient absorption capacity by plants. Previous studies also showed that PGPR can break down organic matter and release mineral nutrients into soil [41]. Soil enzyme activities always showed large differences in the treatments with the application of chemical fertilizer or organic fertilizer, and the biodegradation and transformation of the organic matter contained in BOF could significantly increase microbial metabolic activity, activate soil nutrients and promote the absorption of nutrients by crops [42]. 

Carbon is one of the essential parameters in controlling soil microbial metabolism and prompting plant growth [43]. Application of organic fertilizer or BOF can significantly increase the contents of SOC. Invertase activity is also an important metric for soil productivity and soil carbon cycle metabolic capacity [44]. Furthermore, the results indicated that the application of NJAU4742 increased the carbon-related enzyme activities. With the increase of *Trichoderma* spp., the invertase activity and SOC contents were significantly increased, and significant positive correlations were observed among yields, invertase activity, SOC content and *Trichoderma* spp. quantity. The treatments with BOF application showed a positive correlation between phosphatase activity and AP in both soils. Olander and Vitousek et al. [45] found that the phosphatase activity in barren hilly soils was significantly higher than that in the loamy sandy soil, which is consistent with the results obtained in this study. The negative feedback inhibition phenomenon appeared in different treatments intensively when soil contained a sufficient P level, and the phosphatase activity was significantly reduced because of high nutrient content in the soil, which would weaken the microbial effects. PGPF in the rhizosphere soil transform, mobilize and dissolve nutrients more effectively than those in the bulk soils [46]. Rivera-Cruz et al. [47] also reported that the rhizosphere microbial activity of the reactivation function and the soil microbial community-mediated organic matter turnover and nutrient cycling processes increase the utilization of plant nutrients. Simultaneously, soil organic carriers (i.e., straw, compost, feces) increase the capacity for C, N and P. By contrast, nutrients also affect microbial community structure and activity [48]. 

A tremendous increase of soil enzyme activities was detected in the present investigation, particularly for urease and peroxidase activities, and the possible reason could be that NJAU4742 accelerated the microbiological oxidizing-reducing reactions and the nitrogen metabolism in the soil, which would provide a possible mechanism of plant growth promotion by beneficial microorganisms. Determination of the nitrogen conversion related-enzyme activities could help us to understand the interactions among organic matter, the quantity of *Trichoderma* spp. and soil enzyme activities in the comparison of the abundance and quality of the organic matter and for a high dose of a *Trichoderma* population [49]. The urea use efficiency in the nitrogen cycle and the rate of decomposition in soil are characterized by urease [50], and urease initially catalyzes the hydrolysis of ammonium carbonate in soil by urea hydrolysis, releasing ammonium ions (NH_4_^+^-N) and carbon dioxide: CO(NH_2_)_2_ + H_2_O + 2H^+^ → CO_2_ + 2NH_4_^+^ [51]. This category of PGPR has been reported on widely [5], whereas the values of urease activity obtained in different treatments, particularly in those inoculated with NJAU4742 (58.7 g·g^−1^ dw), tended to be higher than those of other beneficial strains (19.72 g·g^−1^ dw) [52]. These findings suggested that addition of rhizosphere beneficial microbes could significantly promote soil urea conversion efficiency. Thus, urease activity could be a primary indicator with which to evaluate the capacity of nitrogen transformation in different soils.

### 4.3. Soil Microbial Diversity

Soil microbial diversity is one of the critical parameters in plant growth promotion, which influences various biochemical processes, including nutrient acquisition, nitrogen cycling and carbon cycling [53]. Aber et al. [54] have pointed out that the microbial communities in the rhizosphere were hard to change by the introduction of foreign microbes. However, Kampfenkel et al. [55] found that the inoculation of *A. brasilense* Sp6 resulted in changes of the DGGE profile in the rhizospheric community at 15, 30, and 60 days following bacterial inoculation, and the community structure changed even more significantly as plants established themselves and grew. Similarly, Jian et al. [32] investigated the ecological roles of beneficial inoculants and their potential impacts on soil microbial diversity, and their results indicated that the beneficial inoculants could interfere with the soil health and microbial/faunal community composition. In this study, *Trichoderma guizhouense* NJAU4742 were applied into the soil during the pot experiment, and the quantification results showed that the numbers of bacteria, fungi and *Trichoderma* spp. gradually increased with the increasing number of NJAU4742 added into the soil; however, there was no direct evidence as to whether the inoculation of NJAU4742 could also change the microbial diversity in this study, and more in-depth study is necessary in subsequent research. It was a relief that Umadevi et al. [56] found that the functional richness and population dynamics of rhizosphere bacteria, archaea and eukaryotes, as reflected through the selective recruitment of bacteria (*Acidobacteriaceae bacterium* (*p* = 1.24e^−12^), *Candidatus korib-acter versatilis* (*p* = 2.66e^−10^)) and fungi ((*Fusarium oxysporum* (*p* = 0.013), *Talaromyces stipitatus* (*p* = 0.219) and *Pestalotiopsis fici* (*p* = 0.443)), were influenced by the treatment with *T. harzianum*.

## 5. Conclusions

In this study, the amino acid hydrolysates from slaughterhouse wastes could increase the spore production of NJAU4742 due to the rich nutrients and low pH-stress strategy. A new approach of BOF preparation was developed by adding exogenous amino acid hydrolysate (containing 6.9 % free amino acid) to rice straw, which could significantly increase the production of NJAU4742 sporulation in the conditions of initial pH 3.5 and moisture content 75%, under which the production of spores could reach to 2.40 × 10^10^ spore·g^−1^. The effects of solid spawn and *T. guizhouense* NJAU4742-based BOF on pepper growth promotion were evaluated in sandy and mountain soils during two seasons., and the *Trichoderma*-based BOF had considerable effects on the plant growth promotion through the mechanisms of increasing peroxidase, urease and invertase activities and improving soil nutrient use efficiency. Overall, these findings extended our knowledge of the mechanisms of plant growth promotion by BOFs and are anticipated to benefit the development of the utilization of agricultural waste for sustainable agriculture.

## Figures and Tables

**Table 1:** The growth-related traits (shoot height, stem diameter), fruit yield and quality of first season peppers with different treatments in two kinds of soils.

Treatments	Soil Type	Length (cm)	Diameter (mm)	Yield g·pot^−1^	Number Fruit	VC^−^ (mg·kg^−1^)	NO_3_^−^ (mg·kg^−1^)	Protein (g·kg^−1^)
CK	Sandy soil	13.00	2.63	42.52e ± 8.20	3.0c ± 0.32	2.33e ± 0.11	4.34d ± 0.04	55.53d ± 0.25
	Mountain soil	6.36	1.53	21.11e ± 5.79	2.00b ± 0.45	1.70f ± 0.02	5.30f ± 0.01	36.07d ± 1.06
T0	Sandy soil	13.02	2.63	43.46e ± 12.62	3.0ab ± 0.55	2.29e ± 0.13	4.34d ± 0.01	55.38d ± 0.07
	Mountain soil	6.38	1.51	20.45e ± 4.70	2.00b ± 0.32	1.66de ± 0.012	5.14ef ± 0.02	39.21d ± 2.62
T1	Sandy soil	13.62	2.80	45.02e ± 3.86	3.2ab ± 0.37	2.38e ± 0.05	4.23d ± 0.01	56.06d ± 0.14
	Mountain soil	6.44	1.52	20.01e ± 0.58	2.00b ± 0.37	1.77cde ± 0.01	5.32e ± 0.01	37.65d ± 0.31
T3	Sandy soil	15.04	2.93	50.36cd ± 1.51	3.4ab ± 0.25	2.58de ± 0.07	4.23d ± 0.01	57.13cd ± 0.23
	Mountain soil	6.40	1.59	25.34cd ± 3.84	2.00b ± 0.25	1.80cd ± 0.04	4.89d ± 0.03	46.22c ± 0.52
T6	Sandy soil	15.06	2.98	51.45cd ± 4.32	3.4ab ± 0.51	2.72d ± 0.03	4.22d ± 0.02	57.51cd ± 0.06
	Mountain soil	6.46	1.61	29.09cd ± 4.99	2.00b ± 0.51	1.89c ± 0.014	4.85cd ± 0.08	48.48c ± 1.12
AA	Sandy soil	16.94	3.18	55.05bc ± 3.41	3.2ab ± 0.20	3.14c ± 0.13	3.82c ± 0.10	60.03c ± 0.46
	Mountain soil	8.34	1.79	48.72bc ± 3.10	3.00a ± 0.37	2.10b ± 0.09	4.51b ± 0.01	52.06b ± 0.15
AT1	Sandy soil	17.02	3.19	56.29bc ± 6.91	3.4a ± 0.25	3.14c ± 0.02	3.76ab ± 0.05	63.91b ± 0.30
	Mountain soil	8.76	1.81	49.49bc ± 3.05	3.00a ± 0.58	2.15b ± 0.053	4.26a ± 0.07	55.74a ± 1.18
AT3	Sandy soil	17.50	3.41	63.65ab ± 9.23	3.8ab ± 0.37	3.62ab ± 0.03	3.66ab ± 0.01	68.39a ± 1.63
	Mountain soil	9.30	1.90	57.54ab ± 2.79	3.00a ± 0.25	2.21b ± 0.02	4.26a ± 0.15	57.01a ± 0.53
AT6	Sandy soil	18.36	3.52	66.83a ± 4.59	4.2a ± 0.37	3.79a ± 0.04	3.51a ± 0.01	68.05a ± 1.48
	Mountain soil	9.40	2.11	60.04a ± 8.51	3.00a ± 0.49	2.34a ± 0.10	4.10a ± 0.00	58.86a ± 1.14
BT	Sandy soil	16.22	3.11	59.61cd ± 3.41	3.6 ± ab0.40	3.38bc ± 0.19	4.08d ± 0.23	57.23cd ± 0.61
	Mountain soil	9.12	2.02	45.08cd ± 2.68	3.00a ± 0.63	1.77cde ± 0.04	4.68bc ± 0.00	47.37c ± 1.29

Values (means ± SD, *n* = 5) within the same column followed by different letters are significantly different at *p* < 0.05 according to Tukey’s post-hoc test. Treatment: CK, control with chemical fertilizer; T0, 0.01% sterilized fermentation products; T3, 0.03% sterilized fermentation products; T6, 0.06% sterilized fermentation products; AA, 20% amino acid organic fertilizer; AT1, amino acid bio-organic fertilizer with 1% fermentation products; AT3, amino acid bio-organic fertilizer with 1% fermentation products; AT6, amino acid bio-organic fertilizer with 1% fermentation products; BT, a comparable, common biological organic fertilizer.

**Table 2:** The pepper yields of different treatments in two kinds of soil during two seasons.

Treatments	First Season				Second Season			
	Sandy Soil	Increasing Rate	Mountain Soil	Increasing Rate	Sandy Soil	Increasing Rate	Mountain Soil	Increasing Rate
CK	225.63	-	124.65	-	168.68	-	105.57	-
T0	253.98	12.56%	117.52	−5.72%	186.38	10.49%	102.23	−3.16%
T1	259.80	15.14%	140.06	12.36%	175.97	4.32%	109.34	3.57%
T3	299.92	32.93%	164.78	32.19%	219.7	30.25%	118.79	12.52%
T6	320.14	41.89%	174.27	39.81%	256.33	51.96%	160.05	51.61%
AA	319.79	41.73%	182.55	46.45%	216.75	28.50%	215.34	103.98%
AT1	304.84	35.11%	180.92	45.14%	228.94	35.72%	202.95	92.24%
AT3	334.98	48.46%	224.13	79.81%	247.4	46.67%	225.12	113.24%
AT6	343.54	52.26%	230.11	84.60%	300.57	78.19%	260.22	146.49%
BT	245.67	8.88%	188.34	51.10%	280.18	66.10%	214.97	103.63%

“-” No results; F means first season; S means second season.

**Table 3:** The physiochemical characterization of sandy soil during two seasons after the harvest.

Treatments		SOC (g·kg^−1^)	TN (g·kg^−1^)	NH_3_^+^ (mg·kg^−1^)	NO_3_^−^ (mg·kg^−1^)	AP (mg·kg^−1^)	AK (mg·kg^−1^)	pH
**FS**	CK	8.31f ± 0.02	8.75cd ± 0.03	1.34d ± 0.09	297.18a ± 2.16	87.58g ± 0.33	95.333e ± 1.45	7.44
	T0	8.91e ± 0.08	8.78cd ± 0.02	1.21d ± 0.07	261.95ab ± 8.93	87.89fg ± 0.23	96.333de ± 4.84	7.41
	T1	8.93e ± 0.02	8.64d ± 0.16	1.39d ± 0.11	289.30a ± 16.10	86.46fg ± 1.61	104.33cd ± 1.45	7.37
	T3	9.15d ± 0.04	9.15bc ± 0.10	1.89bc ± 0.02	278.06a ± 4.42	91.5cde ± 0.96	109.33bc ± 0.33	7.33
	T6	9.16d ± 0.11	9.31ab ± 0.20	2.06ab ± 0.06	284.57a ± 7.89	96.43ab ± 1.46	116.33ab ± 4.25	7.32
	AA	9.46c ± 0.02	9.35ab ± 0.21	1.82c ± 0.04	184.43bc ± 3.50	90.24efg ± 2.64	106.66c ± 0.67	7.39
	AT1	9.90b ± 0.06	9.52ab ± 0.10	2.02bc ± 0.05	151.51c ± 4.68	95.28abc ± 1.02	112.66bc ± 0.88	7.40
	AT3	9.99b ± 0.07	9.37ab ± 0.14	2.11ab ± 0.03	157.95c ± 5.24	96.40ab ± 1.78	116.00ab ± 3.00	7.35
	AT6	10.19a ± 0.09	9.66a ± 0.15	2.24a ± 0.07	140.20c ± 3.81	97.98a ± 1.19	122.00a ± 4.04	7.33
	BT	10.08f ± 0.04	9.48ab ± 0.23	1.92bc ± 0.09	183.79bc ± 9.93	92.81a ± 0.57	117.00ab ± 2.52	7.32
**SS**	CK	7.34e ± 0.02	0.81bc ± 0.01	2.39b ± 0.14	140.71a ± 1.90	7.68b ± 0.28	195.66ab ± 7.21	7.41
	T0	7.84d ± 0.02	0.78c ± 0.01	2.34ef ± 0.08	133.04a ± 4.51	7.45b ± 0.27	185.66bcd ± 7.01	7.47
	T1	7.60d ± 0.02	0.76de ± 0.00	2.44d ± 0.22	139.59b ± 5.48	6.54b ± 0.25	180.33bcd ± 4.63	7.49
	T3	8.31c ± 0.08	0.75de ± 0.00	2.64e ± 0.41	123.93b ± 4.69	6.15b ± 0.42	168.00e ± 9.17	7.53
	T6	8.35c ± 0.04	0.84b ± 0.01	3.09cd ± 0.11	108.38c ± 1.60	6.91b ± 0.16	206.00a ± 1.53	7.51
	AA	9.33b ± 0.23	0.79c ± 0.01	2.42g ± 0.14	32.53d ± 1.22	13.06a ± 0.04	172.66e ± 1.58	7.68
	AT1	9.10a ± 0.35	0.81de ± 0.01	3.40c ± 0.30	30.04de ± 1.85	14.69a ± 0.62	168.00e ± 0.58	7.67
	AT3	9.86b ± 0.02	0.74b ± 0.01	4.70f ± 0.09	31.27de ± 0.37	12.92a ± 0.18	179.00abc ± 5.78	7.66
	AT6	10.04b ± 0.10	0.82a ± 0.04	4.84ef ± 0.17	19.78e ± 0.93	11.97a ± 0.13	194.3de ± 4.33	7.62
	BT	9.90a ± 0.08	1.05bc ± 0.01	4.58a ± 0.41	95.18c ± 8.83	15.06a ± 0.61	205a ± 2.08	7.67

* FS: the first season; SS: the second season. SOC means soluble organic carbon; TN means total nitrogen; AP means available phosphate; AK means available potassium. Values (means ± SD, *n* = 5) within the same row followed by different letters are significantly different at *p* < 0.05 according to Tukey’s post-hoc test. The “a” letter represents the ANOVA results for the different treatments for peppers.

**Table 4:** The physiochemical characterization of red soil during two seasons after the harvest.

Treatments		SOC (g·kg^−1^)	TN (g·kg^−1^)	NH_3_^+^ (mg·kg^−1^)	NO_3_^−^ (mg·kg^−1^)	AP (mg·kg^−1^)	AK (mg·kg^−1^)	pH
**FS**	CK	7.34e ± 0.02	0.84a ± 0.14	170.71a ± 1.90	0.78de ± 0.00	6.01d ± 0.40	175de ± 2.08167	7.41
	T0	7.84d ± 0.02	0.84a ± 0.08	133.04c ± 4.51	0.78de ± 0.01	6.12d ± 0.09	175.6de ± 1.67	7.48
	T1	7.60de ± 0.02	0.85ab ± 0.22	139.59c ± 5.48	0.76de ± 0.00	6.38d ± 0.10	177.66d ± 2.85	7.49
	T3	8.31c ± 0.08	0.90b ± 0.17	123.93c ± 4.69	0.80d ± 0.01	5.99d ± 0.29	188.00b ± 4.16	7.44
	T6	8.35c ± 0.04	1.07c ± 0.11	158.38b ± 1.60	0.84c ± 0.01	6.91d ± 0.16	206.00a ± 1.53	7.41
	AA	8.97b ± 0.12	1.57d ± 0.14	50.04e ± 10.22	0.79d ± 0.00	12.02c ± 0.43	179.33cd ± 2.33	7.47
	AT1	9.39a ± 0.06	1.64e ± 0.29	31.27f ± 1.84	0.83bc ± 0.01	12.25c ± 0.29	174.66de ± 3.18	7.47
	AT3	9.44a ± 0.02	1.74ef ± 0.09	19.78f ± 0.37	0.89a ± 0.00	12.57bc ± 0.17	185.66bc ± 2.85	7.43
	AT6	9.63a ± 0.11	1.95f ± 0.17	22.53f ± 0.93	0.91a ± 0.02	13.39a ± 0.21	207.66a ± 3.18	7.39
	BT	9.57a ± 0.28	1.30cd ± 0.12	95.18d ± 8.83	0.84b ± 0.02	12.06c ± 0.81	205.00a ± 2.08	7.41
**SS**	CK	8.36d ± 0.18	0.79e ± 0.00	0.86d ± 0.15	324.41a ± 1.90	5.58f ± 0.08	124.16e ± 1.74	7.41
	T0	8.51cd ± 0.02	0.81cd ± 0.00	0.85d ± 0.01	304.40ab ± 11.26	5.43fg ± 0.30	126.38e ± 1.47	7.47
	T1	8.47cd ± 0.06	0.80e ± 0.00	0.87d ± 0.10	289.03b ± 15.35	5.87f ± 0.04	125.83e ± 1.44	7.44
	T3	8.65cd ± 0.06	0.80b ± 0.02	0.95d ± 0.10	265.91bc ± 2.44	6.21de ± 1.85	133.88d ± 2.42	7.47
	T6	8.62d ± 0.04	0.84bc ± 0.01	1.05bc ± 0.10	219.57d ± 7.88	6.25d ± 0.05	141.38bc ± 2.00	7.44
	AA	9.51b ± 0.10	0.84b ± 0.00	1.56c ± 0.11	30.37f ± 9.93	12.96c ± 0.17	138.61cd ± 1.00	7.47
	AT1	10.02ab ± 0.02	0.84ab ± 0.01	1.63bc ± 0.11	87.62f ± 9.93	13.54ab ± 0.13	136.94cd ± 1.00	7.46
	AT3	10.31a ± 0.06	0.92b ± 0.00	1.78ab ± 0.08	83.12f ± 1.40	13.69ab ± 1.01	145.83b ± 1.73	7.42
	AT6	10.36a ± 0.04	0.94a ± 0.01	1.96a ± 0.08	81.99g ± 0.98	13.88ab ± 0.89	157.72a ± 1.95	7.48
	BT	9.37bc ± 0.12	0.94a ± 0.01	1.32d ± 0.05	114.12e ± 2.78	13.09a ± 0.42	136.38cd ± 5.05	7.45

* FS means first season; SS means second season; SOC means soluble organic carbon; TN means total nitrogen; AP means available phosphate; AK means available potassium. Values (means ± SD, *n* = 5) within the same row followed by different letters are significantly different at *p* < 0.05 according to Tukey’s post-hoc test. The “a” letter represents the ANOVA results for the different treatments for pepper.

## Supplementary Materials

Supplementary materials can be found at https://www.mdpi.com/2076-2607/8/9/1296/s1. Table S1. The amino acid concentrations of the liquid amino acid solution. Table S2. The physiochemical properties of different materials used in this study. Table S3. The description of different treatments designed in this study. Table S4. Primers of fluorescent quantitative PCR used in this study.
