# Supplementary material for: The Growth Promotion of Peppers (Capsicum annuum L.) by Trichoderma guizhouense NJAU4742-Based Biological Organic Fertilizer: Possible Role of Increasing Nutrient Availabilities
Source: Microorganisms. 2020 Aug 25;8(9):1296. doi: 10.3390/microorganisms8091296 (PMC7565307; doi:10.3390/microorganisms8091296)
Supplement: Supplementary file 1 [file microorganisms-08-01296-s001.pdf]

Article

The Growth Promotion of Peppers (*Capsicum annuum L*) by *Trichoderma guizhouense* NJAU4742-Based Biological Organic Fertilizer: Possible Role of Increasing Nutrient Availabilities

Qiumei Liu, Xiaohui Meng, Tuo Li, Waseem Raza, Dongyang Liu* and Qirong Shen
Jiangsu Provincial Key Lab of Solid Organic Waste Utilization, Jiangsu Collaborative Innovation Center of Solid Organic Wastes, Educational Ministry Engineering Center of Resource-saving fertilizers, Nanjing Agricultural University, Nanjing 210095, Jiangsu, Peoples Republic of China; 2016203055@njau.edu.cn (Q.L.), 2015203032@njau.edu.cn (X.M.), 2018103144@njau.edu.cn (T.L.), 2018103120@njau.edu.cn (W.R.), shenqirong@njau.edu.cn (Q.S.)
*Correspondence: liudongyang@njau.edu.cn; Tel: +86 25 84396853; Fax: +86 25 84396853

Table S1. The amino acid concentrations of the liquid amino acids solution.

Amino acid name	RT	Height	Peak area	Concentration（$mg \cdot mL^{-1}$）
Asp	4.767	181204	2888603	11.29
Thr	5.44	127691	2121904	2.39
Ser	6.033	247321	4134286	6.36
Glu	6.72	291618	5912522	10.44
Gly	9.687	258359	4686773	2.09
Ala	10.373	104705	2519495	7.9
Cys	12.32	101980	1065874	4.15
Val	12.793	146755	2071069	1.38
Met	13.933	20804	438836	1.79
Ile	16.193	43543	1242722	0.82
Leu	17.313	95924	3391999	0.91
Tyr	18.52	23103	1138372	3.12
Phe	20.667	50905	1046558	1.66
Lys	23.527	137816	1844916	3.41
His	25.627	30946	554956	2.03
Arg	29.687	90329	2424415	0.81
Pro	7.467	24772	391232	5.22
Total N	—	—	—	3.51

Table S2. The physiochemical properties of different materials used in this study.

Treatments	pH	Total N ($g \cdot kg^{-1}$)	Total P ($g \cdot kg^{-1}$)	Total K ($g \cdot kg^{-1}$)
Amino acid organic fertilizer	6.74±0.21	19.80±0.210	10.18±0.21	12.11±1.05
Rice straw	7.84±0.11	2.77±0.02	1.63±0.07	11.19±1.01
Amino acid solution	0.5±0.05	42.16±0.41	—	—
Fermentation products	5.73±0.17	22.35±0.31	2.43±0.05	11.81±0.81
Mountain red soil	7.37±0.21	0.84±0.05	0.81±0.01	0.113±0.04
Sandy soil	7.51±0.43	8.761±0.41	0.87±0.01	0.254±0.02

"—" means not detected

Table S3. The description of different treatments designed in this study.

Treatments	Detailed definition	Fertilization			
		Chemical fertilizer (g)			Organic fertilizer (g·pot^{-1})
		Urea (g·pot^{-1})	Superphosphate (g·pot^{-1})	Sulphate Potash (g·pot^{-1})	
CK	Chemical fertilizer	1.41	1.68	1.23	—
T0	Fermentation of *Trichoderma* (0.01% sterilization)	1.41	1.68	1.23	—
T1	Fermentation of *Trichoderma* (0.01%)	1.41	1.68	1.23	—
T3	Fermentation of *Trichoderma* (0.03%)	1.41	1.68	1.23	—
T6	Fermentation of *Trichoderma* (0.06%)	1.41	1.68	1.23	—
AA	20% amino acid organic fertilizer(v/w)	—	—	—	30.00
AT1	20% amino acid organic fertilizer (v/w) + fermentation of *Trichoderma* (1%)	—	—	—	30.30
AT3	20% amino acid organic fertilizer (v/w) + fermentation of *Trichoderma* (3%)	—	—	—	30.90
AT6	20% amino acid organic fertilizer (v/w) + fermentation of *Trichoderma* (6%)	—	—	—	31.80
BT	Common biological organic fertilizer	—	—	—	30.00

Table S4. Primers of fluorescent quantitative PCR used in this study.

Microbial species	Genome location or name	Primer	Sequence
Bacterial	515F/907R	Forward primer	ACTCCTACGGGAGGCAGCAG
		Reverse primer	ATTACCGCGGCTGCTGG
Fungi	ITS5/ITS4	Forward primer	TCCGTAGGTGAACCTGCGG
		Reverse primer	CGCTGCGTTCTTCATCG
T. NJAU4742	*T37_S00007:2096560-2099766*	Forward primer	GGATCATTACCGAGTTTACAACTCC
		Reverse primer	CCGTTGTTGAAAGTTTTGATTCATTT
